# Recent Advances in the Management of Relapsed and Refractory Peripheral T-Cell Lymphomas

**DOI:** 10.3390/jpm12060964

**Published:** 2022-06-13

**Authors:** Zachary Braunstein, Miguel Ruiz, Walter Hanel, Polina Shindiapina, John C. Reneau, Jonathan E. Brammer

**Affiliations:** 1 Wexner Medical Center, Department of Internal Medicine, The Ohio State University, Columbus, OH 43210, USA; zachary.braunstein@osumc.edu (Z.B.); miguel.ruiz@osumc.edu (M.R.); 2 James Comprehensive Cancer Center, Division of Hematology, Department of Internal Medicine, The Ohio State University, Columbus, OH 43210, USA; walter.hanel@osumc.edu (W.H.); polina.shindiapina@osumc.edu (P.S.); john.reneau@osumc.edu (J.C.R.)

**Keywords:** T cell, lymphoma, leukemia, peripheral T-cell lymphoma, peripheral T-cell lymphoma not otherwise specified, angioimmunoblastic T-cell lymphoma, adult T-cell lymphoma, therapeutics, relapsed, refractory, novel therapy

## Abstract

Peripheral T-cell lymphomas (PTCLs) are a group of heterogeneous lymphomas with poor overall prognosis, particularly in the setting of relapsed/refractory PTCL. Given the limited efficacy of current therapies, several different novel therapies encompassing multiple different mechanisms of action have been evaluated for relapsed and refractory PTCLs. In this review, we explore the current standard of care for relapsed/refractory PTCL, and evaluate in depth novel and emerging therapies, their scientific basis, and current trials for relapsed/refractory PTCL.

## 1. Introduction

Peripheral T-cell lymphomas (PTCLs) are a group of rare, heterogeneous non-Hodgkin lymphomas (NHLs) with an aggressive disease course. In the most recent World Health Organization (WHO) classification, 27 unique types of PTCLs were described, including several provisional entities [1]. PTCLs account for approximately 5–10% of NHLs in western countries [2,3]. The incidence does vary geographically, and the differences were demonstrated by the International T-cell Lymphoma Project [2]. In North America, the most common PTCL subtypes are peripheral T-cell lymphoma, not otherwise specified (PTCL-NOS) (34%); angioimmunoblastic T-cell lymphoma (AITL) (16%); anaplastic lymphoma kinase (ALK)-negative anaplastic large-cell lymphoma (ALCL) (16%); and ALK-positive ALCL (8%). These are largely the focus of this review. In Europe, the distribution is very similar, while in Asia, the most common subtypes are adult T-cell leukemia/lymphoma (ATLL), natural Killer/T-cell lymphoma (NKTCL), and PTCL-NOS [2,4]. A further breakdown of the geographic distribution can be seen in Table 1.

While the goal of frontline therapy in PTCL is the attainment of long-term remission or cure, the prognosis of patients with PTCL is generally unfavorable, with 5-year overall survival (OS) rates around 30–35%, aside from patients with ALCL [5,6,7,8].

The standard backbone of therapy is with cyclophosphamide/doxorubicin/vincristine/prednisone (CHOP), or CHOP-associated regimens, such as CHOP + etoposide (CHOEP) or cyclophosphamide/doxorubicin/prednisone (CHP) + Brentuximab vedotin (BV) (in CD30-positive patients) [2,4,5]. Recently, the pivotal ECHELON-2 trial, a randomized phase III study evaluating the use of BV in the frontline treatment of CD30-positive PTCL, showed a statistically significant improvement in both progression-free survival and OS versus standard CHOP therapy [9]. For patients that do achieve a response, most fit patients receive consolidation with carmustine, etoposide, cytarabine, and melphalan (BEAM) followed by autologous stem-cell transplant (ASCT) [10]. While BEAM is the most common regimen used for consolidation, other regimens, including total body irradiation + cyclophosphamide (TBI-Cy) and carmustine, cyclophosphamide, and etoposide (BCV), have also been used [11,12,13]. Due to the rarity and heterogeneity of the disease and its treatment, no randomized control trials have been, or likely will be, conducted for the consolidation of PTCL. However, given the aggressive nature of PTCL relapses, many experts recommend consolidative transplant based upon retrospective and prospective trials. This is also in line with the NCCN guidelines [10,13,14,15]. While patients can often attain a response with frontline therapy, unfortunately, a majority of patients with PTCL progress, and the outcomes of patients with relapsed/refractory (r/r) PTCL are dismal, with a median survival of 4 months [4,16,17]. This review focuses on the management of patients with PTCL, with a specific emphasis on emerging novel therapies in the setting of r/r disease. 

## 2. Current Standard of Care for Relapsed/Refractory PTCL

The current approach for the treatment of r/r PTCL is divided into two primary categories: salvage therapy in preparation for stem-cell transplant (SCT) (typically allogeneic) or single-agent treatment, either for palliative purposes or as bridging therapy in hopes of proceeding to a potentially curative transplant. For the majority of patients with r/r PTCL who are eligible for SCT, the goal of salvage therapy is to attain clinical remission in order to proceed to allogeneic SCT (allo-SCT) [10]. If a patient is unfit for allo-SCT, or if they have relapsed ALCL with clear chemo-sensitivity and a CR to salvage therapy, autologous transplant can be considered [10]. For chemotherapy-intensive regimens, age may lead to a preferential choice of a single-agent regimen, such as single-agent gemcitabine or bendamustine, or the use of novel agents, which often have a lower toxicity profile. With novel agents, age should not be a limiting factor. For all patients, the chosen regimen should be individually chosen based on the patient’s age, performance status, disease status, comorbidities, and organ function. Given the poor prognosis of r/r PTCL and the low response rates to current therapies, attention has been given to providing targeted therapies in hopes of improving response rates and survival. A breakdown of the current standard of care can be seen in Table 2.

### 2.1. Romidepsin

The term epigenetic is defined as an alteration in the phenotype of cells without changes in the sequence of DNA. Chromatin reorganization is a common epigenetic phenomenon in malignant cells and plays an important role in the pathogenesis and survival of PTCL cells [23,24]. Both AITL and PTCL-NOS have been shown to have relatively high rates of mutations in epigenetic regulators TET2, DNMT3A, IDH2, and KMT2D [25]. Other epigenetic modifiers that have been found to be dysregulated in PTCL include the histone acetyl transferase (HAT) and histone deacetylase (HDAC) families, which play opposing roles in the transfer and removal of acetyl groups from histones, respectively (reviewed in [26]). HDACs can enhance pro-survival pathways in PTCL, including c-myc, PI3K/Akt, and NF-kappaB [27,28,29]. Conversely, HDACs have also been shown to suppress tumor suppressor genes such as p53 and BIM [30,31]. Thus, HDACs have surfaced as a target for anti-PTCL therapies. 

Romidepsin is a natural product obtained from bacterium *Chromobacterium violaceum*, which undergoes reduction in cells to form a free thiol group and binds to a Zn atom of HDACs, thus inhibiting their deacetylase activity with specificity for class I HDACs (1, 2, 3, and 8); this was FDA-approved for the treatment of PTCL in 2011 (for commercial reasons, FDA approval was withdrawn in 2021, though it remains a standard agent in the NCCN guidelines and is commercially available in the US) [13,32]. Romidepsin as a monotherapy was investigated in two phase II studies of patients with r/r PTCL. In the first study, patients received 14 mg/m^2^ on D1, -8, and -15 of 28-day cycles. This trial enrolled patients with various subtypes of PTCL (*n* = 47), and an overall response rate (ORR) of 38% (CRR of 18%) with a median duration of response of 8.9 months was seen [19]. In the patients that achieved a CR, the median DoR was 29.7 months. In a pivotal study in R/R PTCL (*n* = 130), an ORR of 25% (CRR of 15%) with a median PFS of 29 months and duration of response (DOR) of 28 months was observed [33,34]. Importantly, amongst the patients with angioimmunoblastic T-cell lymphoma in this study, response duration was long, with a median DOR of 38 months. Romidepsin remains an important standard option for patients with relapsed PTCL, particularly in those with AITL, showing a 30% ORR and 44% disease control (ORR + stable disease (SD)), as well as in PTCL-NOS patients, with a 29% ORR and 49% disease control [33,34]. In patients with a CR, a ‘maintenance’ romidepsin strategy is employed by many clinicians, with decreasing frequency with serial imaging over 6–12 months. Patients are typically given full-dose romidepsin for 6 months, and if a CR persists, the frequency of romidepsin is reduced to a more practical every-2-week frequency and then to a schedule of every 3 or 4 weeks over time, with serial imaging to ensure the maintenance of remission. Romidepsin is increasingly used in combination with other agents to improve response rates, and these combinations are discussed in greater detail below.

### 2.2. Belinostat

Belinostat is another HDAC inhibitor with less specificity than romidepsin, inhibiting HDACs in class I, II, and IV. The BELIEF study was a registration, direct phase II trial of belinostat in r/r PTCL in which belinostat was administered IV on days 1–5 of a 21-day cycle [35]. Of 129 patients, 25.8% of patients achieved a response, with 10.8% experiencing a CR. The median PFS was 1.6 months with a DOR of 13.6 months. The subset of patients with AITL (*n* = 22) had a higher ORR than the others, at 45.5%. These results led to the approval of belinostat in patients with R/R PTCL, irrespective of the number and types of prior therapies. Recently, a small phase I study combined Belinostat with frontline CHOP therapy with a dose escalation portion (*n* = 11) and expansion cohort (*n* = 12) [36]. The MTD of belinostat with CHOP was found to be 1000 mg/m^2^ on days 1–5, which was further evaluated in the expansion cohort. In the expansion cohort, an ORR of 86% and a CRR of 71% were obtained, which were overall better than the historical response rates in PTCL patients. In patients with AITL, the ORR of belinostat with CHOP was found to be 89%, and it was 90% in patients with bone marrow involvement [36]. This preliminary signal would need validation in randomized studies, especially given the negative results of a recent randomized trial comparing romidepsin–CHOP to CHOP alone [37]. Combination strategies with Belinostat are still an open area of investigation in r/r PTCL.

### 2.3. Pralatrexate

Pralatrexate, a folate antagonist similar to methotrexate, was the first drug to be FDA-approved for the treatment of relapsed/refractory PTCL in 2009. In the initial phase I trial, an ORR of 54% was observed, with a 23% PR and a 31% CR [38]. These data led to the PROPEL trial, a phase II, single-arm, open-label, international multicenter study. The results of this trial demonstrated an ORR of 29% (11% CR, 18% PR), while providing a median DoR of 10.1 months and an OS of 14.1 months [20] in responding patients. Similar results have also been seen in several follow-up studies [39,40]. Importantly, patients receiving pralatrexate must be pre-medicated with vitamin B12, folic acid, and leucovorin in order to prevent mucositis and severe cytopenias [41]. This agent has also been explored in combination with romidepsin (see below). Pralatrexate can be an effective therapy for patients with relapsed/refractory PTCL, when administered with proper pre-medications, but due to the toxicities inherent to the drug, it is frequently used after romidepsin.

### 2.4. Bendamustine

Bendamustine is a purine analogue with an alkylating group that has been found to be effective in several indolent hematologic malignancies. In the phase II BENTLY trial, bendamustine was investigated as a single agent with 90% AITL and PTCL-NOS patients. Bendamustine was administered at 90–120 mg/m^2^ on days 1–2 of a 21 day cycle, for up to six cycles, which resulted in an ORR of 50% (28% CR, 22% PR) and a mean DoR of 3.5 months [21]. Additionally, combinations involving bendamustine have been studied and are looked at below in this review. While remission with bendamustine is short, given its good tolerability, this agent is a viable option to serve as a bridge to allo-SCT. It may also be of benefit to patients for whom combination chemotherapy is not recommended, such as elderly patients or patients with poor performance status.

### 2.5. Gemcitabine

Gemcitabine is a pyrimidine antimetabolite that has been used in multiple types of aggressive lymphomas. In r/r PTCL, it has been investigated as a single agent and combination therapy. As single therapy, the ORR was demonstrated to be 50–70% [18,42]. In one study gemcitabine was shown to have an ORR of 51% (CR of 23% and 28%) and in a 10-year update, seven of nine CR patients were found to have continuous complete response [18,42]. It has also been investigated in combination therapies to improve response [43], such as in a small study from London that showed an ORR of 69% (CR of 19%) to Gemcitabine, Cisplatin, and methylprednisolone [44].

### 2.6. ICE: Ifosfamide + Carboplatin + Etoposide

ICE (Ifosfamide, carboplatin, and etoposide) is combination therapy typically used as a salvage regimen for HDT/ASCT in relapsed/refractory PTCL. In a retrospective analysis, 14 patients with BV-ICE had an ORR of 29% (CR of 14%), but few attained a sustained response [45]. In another retrospective analysis of 30 patients (16 DexaBEAM, 15 ICE), the ORRs were found to be 69% vs. 20%, respectively [22]. This combination remains an important option, particularly for patients with rapidly progressing PTCL, and it is the recommended frontline combination for patients with hepatosplenic T-cell lymphoma [46]. In patients that are elderly and have a poor performance status, combination chemotherapy regimens, such as ICE, may be of limited utility due to the toxicity associated with the regimen.

### 2.7. Brentuximab Vedotin

CD30 is a member of the tumor necrosis factor superfamily and is predominantly expressed on activated B, T, and NK cells. It is universally and diffusely expressed in anaplastic large-cell lymphoma (ALCL) but is also expressed to a variable degree by other subtypes of PTCLs and CTCLs [47,48]. Previous work suggests that CD30 expression has prognostic significance, especially in PTCL-NOS [49], but other data contradict these findings [50].

Brentuximab vedotin (BV) is an anti-CD30 antibody conjugated with anti-microtubule agent monomethyl auristatin E and is typically dosed at 1.8 mg/kg every 21 days. It was initially approved by the FDA for relapsed and refractory ALCLs based on a pivotal phase II trial showing an objective response rate (ORR) of 86% and a complete response rate (CRR) of 57% with a median duration of response (DOR) of 12.6 months [51]. When broken down by ALK positivity, patients with ALK+ ALCL had an ORR and a CR of 88% and 52%, which was similar to ALK-ALCL patients with an ORR and a CR of 81% and 69%. A long-term follow-up (median of 6 years) demonstrated durable remission, with median OS not being reached in the included trial population [52]. While 5-year follow-up data did not reach median OS nor PFS, 16 patients did remain in remission, with only half receiving consolidative SCT [52]. Among all patients treated in this trial and another evaluating the use of BV in relapsed and refractory classical Hodgkin lymphoma, the most common adverse events reported were neutropenia, peripheral sensory neuropathy (57%), fatigue, nausea, anemia, upper respiratory infection, diarrhea, pyrexia, rash, thrombocytopenia, cough, and vomiting. Importantly, 91% of patients who had experienced peripheral neuropathy on the trial showed improvement or resolution at the long-term follow-up [52]. A planned subset analysis of a phase II multicenter study evaluating the use of BV in CD30-positive non-Hodgkin-lymphoma PTCL demonstrated an ORR of 33% in PTCL-NOS patients and an ORR of 54% in AITL patients [53,54]. In an additional clinical trial with 23 patients with r/r PTCL that had CD30 expression, an ORR of 30.4% was reported (17.4% CR) [55]. During this study, grade III adverse events (SAEs) included: thrombocytopenia (44%), neutropenia (17%), and lung infection/pneumonia (26%). Single-agent BV remains the standard of care for r/r ALCL in patients who did not receive prior BV with up-front therapy. Further, for patients with CD30+ disease, BV should be used before chemotherapy or HDACs. 

## 3. Emerging Novel Agents for Relapsed/Refractory PTCL

### 3.1. Duvelisib

Duvelisib is a dual inhibitor of phosphatidylinositol 3-kinase (PI3K)-δ and PI3K-γ that has been evaluated in large numbers of patients with r/r PTCL [56]. As it inhibits both the δ and the γ PI3K isoforms, it was postulated that Duvelisib not only inhibits the proliferation of lymphoma cells by inhibiting PI3K-δ but also inhibits immune-suppressive M2 tumor-associated macrophages, leading to increased CD8+ cytotoxic T-lymphocyte activation by inhibiting PI3K-γ [56,57].

In a phase I study evaluating duvelisib in a range of lymphomas, duvelisib was found to be effective in patients with PTCL, with an ORR of 50%, with three complete responses [56]. These results led to the phase II, prospective, open-label Primo trial evaluating single-agent duvelisib in patients with r/r PTCL. In this study, patients were first enrolled in a dose-optimization phase, comparing 75 mg twice daily versus 25 mg twice daily of duvelisib [58]. In the dose-optimization phase, the ORR was noted to be 54% in the 75 mg twice daily cohort, versus 35% in the 25 mg twice daily cohort. As a result of this, 75 mg twice daily for two months followed by 25 mg twice daily was determined to be the treatment dose for the expansion phase. To date, the Primo trial has completed the accrual of the expansion phase, though only the results of the planned interim analysis have been presented. In total, 78 reported patients with r/r PTCL were included in the most recent report, with a median of three lines of prior therapy. The ORR by independent assessment was 50%, with a 32% CR rate [58]. Specifically, AITL patients showed a 66.7% ORR with a 47.6% CR rate, and PTCL-NOS patients showed a 52.4% ORR with a 28.6% CR rate [58]. A total of 19.2% of patients stopped treatment due to adverse events, with the most common being grade 3 or greater AEs: neutropenia (38.5%), ALT/AST increase (24.4%, 21.8%), rash (7.7%), and sepsis (6.4%). These results represent the best response rates in r/r non-ALCL PTCLs reported to date.

As a result of these promising data, duvelisib has been added to the NCCN guidelines as another recommended agent (category 2A) in r/r PTCL [13]. Patients with ALCL must fail BV prior to moving forward with duvelisib. While the impressive response rates need to be confirmed in the final results of the Primo trial, this drug should be considered the preferred option for patients with r/r PTCL without CD30 expression. Since nearly 20% of the patients reported thus far discontinued therapy due to adverse events, the careful monitoring of liver function, infection, and other adverse events is needed with this agent.

### 3.2. Valemetostat

Valemetostat is a dual inhibitor of the enhancer of zeste homolog 2 (EZH2) and 1 (EZH1) that functions to down-regulate gene expression by attaching methyl groups to histone H3 on lysine 27 (H3K27me3) [59]. Since epigenetic dysregulation is a hallmark of T-cell lymphomas, valemetostat is thought to increase the gene expression of pro-apoptotic and tumor suppressor genes by altering histone methylation. Valemetostat demonstrated significant success in a phase II trial in Japan in 25 patients with r/r adult T-cell leukemia/lymphoma, with an ORR of 48% and a 20% CR [59]. Subsequently, a phase I dose-escalation trial was conducted in the US and Japan, in 78 patients with r/r PTCL or ATLL [60]. In that study, 45 patients with r/r PTCL were treated with several doses of valemetostat, and an ORR of 55.6% was observed, including 11/45 (24%) CRs. Responses were observed across subtypes of PTCLs; specifically, AITL patients had an ORR of 70.6%, and PTCL-NOS patients had an ORR of 47.6% [60]. Significant side effects were observed, including thrombocytopenia (59%), dysgeusia (51.3%), anemia (37%), neutropenia (35%), alopecia (32%), and decreased WBC (31%). The phase II dose was selected to be 200 mg/day of valemetostat and is due to be tested in the ongoing multi-center, phase II setting in the VALENTINE-PTCL01 study of 176 patients [61]. This drug is not yet available on the market, and the initial results need to be confirmed in the multi-center phase II study, but early results suggest it to be a potentially effective agent in r/r PTCL. Where available, patients should be considered for this important trial (NCT04703192).

### 3.3. MEDI-570

An important subset of T-cell lymphomas are lymphomas of CD4+ T-follicular helper (TFH) origin, whose proliferation is thought to lead to the development of angioimmunoblastic T-cell lymphoma (AITL), PTCL-FH type, and some follicular lymphomas and cutaneous T-cell lymphomas (CTCLs). The inducible T-cell costimulatory (ICOS) protein is highly expressed in these lymphomas, making it an enticing target for novel therapies [62]. An anti-ICOS monoclonal antibody, MEDI-570, was developed to bind and eliminate ICOS-expressing cells, with pre-clinical efficacy in in vivo models [63]. In a phase I NCI study (NCI-9930), MEDI-570 administered every 3 weeks demonstrated efficacy in heavily pre-treated patients with T-cell lymphoma (median of 7.5 prior treatments) [62]. In total, 11/17 (65%) patients had a clinical response, with 4 experiencing partial remission, 7 showing SD, and 2 patients remaining on treatment [62]. All of the patients that had a response were patients that had AITL (*n* = 12). No patients that had PTCL-NOS (*n* = 3) or CTCL (*n* = 2) showed a response to treatment. The treatment was tolerated well overall, with the most common grade 3/4 adverse events being observed: CD4 T-cell decrease (as expected) in 12%, hypophosphatemia (6%), and infusion reactions and thrombocytopenia only occurring in 6% of patients each. This drug is in early development, but early response rates in a highly refractory setting make it a potentially important agent for patients with PTCLs of TFH origin. A breakdown of single agent investigational drugs can be further seen in Table 3. 

## 4. Combination Therapies in Relapsed/Refractory PTCL

Information regarding combination therapies can be seen in Table 4.

### 4.1. Bendamustine Combination Treatments

#### 4.1.1. BCD: Bendamustine + Carboplatin + Dexamethasone

In the phase II BENCART trial, 28/30 patients with ALCL (14%), AITL (25%), and fPTCL-NOS (50%) received a combination of bendamustine, carboplatin, and dexamethasone (BCD) to increase the efficacy of bendamustine. After two cycles, an ORR of 54% (CR of 19%, PR of 35%) was seen. Five patients were eligible for ASCT, while the remainder received four additional cycles, which resulted in a 54% ORR (CR of 29%, PR of 25%). BCD combination seems to be particularly effective in AITL [73]. 

#### 4.1.2. Bendamustine + Brentuximab Vedotin

A recent multicenter, retrospective study evaluated the combination of Bendamustine and BV in 82 patients with r/r PTCL [69]. The ORR was 71%, with 51% of patients achieving a CR. The patients that achieved a CR had a median DoR of 15.4 months, but there was a significant difference in DoR in patients that did go on to SCT versus those who did not (not reached vs. 8.4 months). More than half (59%) of patients had grade 3–4 toxicities, and these were mainly hematologic in nature, but further specifics were not provided.

### 4.2. Romidepsin Combinations

Since the demonstration of single-agent activity and FDA granting accelerated the approval of romidepsin in 2011, romidepsin has been combined with multiple other therapies in trials of r/r PTCL.

#### 4.2.1. Romidepsin + 5-Azacytidine

The DNA methyltransferase inhibitor 5-azacytidine (5-aza) has shown synergy with romidepsin in T-cell lymphoma (CTCL) cell lines and primary samples with increased expression of tumor suppressor RhoB [74]. In an initial phase I dose-finding study in which 5-aza priming was started one week prior to the start of romidepsin administration [75], a minimum tolerated dose (MTD) of 5-aza of 300 mg was given on days 1–14, with romidepsin being given on days 8, 15, and 22, of a 35-day cycle. This led to the initiation of a phase II study on r/r PTCL (NCT01998035), and the preliminary results (*n* = 25) were recently reported at ASH 2021 [70]. An ORR of 61% and a CRR of 42% were seen, with patients with a T-follicular helper phenotype (t-TFH) showing an impressive ORR of 80% and a CRR of 67% [70], likely due to the higher epigenetic mutation burden of AITL in this subset as discussed above. At a median follow-up of 13.5 months, median PFS and DOR were 8.0 months and 20.3 months, respectively. Myelosuppression was the most frequent grade 3–4 toxicity (thrombocytopenia, 48%; neutropenia, 40%; and anemia, 14%). These impressive results show that 5-aza + romidepsin may be a highly effective combination epigenetic therapy in PTCL, particularly for patients with a t-TFH phenotype, but it is still early for definitive results, and further recruitment and follow-ups are necessary. A randomized phase IIB clinical trial comparing romidepsin vs. romidepsin + 5-azacytidine is currently underway (NCT04747236) to definitively determine the effect of this promising combination.

#### 4.2.2. Romidepsin + Pralatrexate

Romidepsin has also been studied in combination with pralatrexate in a phase I study [71]. An every-other-week schedule for each (pralatrexate at 25 mg/m^2^, romidepsin at 12 mg/m^2^) was determined to be the recommended phase II dosing. In 14 patients with PTCL, an ORR of 71% and CRR of 28% was reported. The most common grade 3–4 toxicities were anemia (29%), thrombocytopenia (28%), oral mucositis (14%), and febrile neutropenia (14%). This combination requires future studies, but the promising ORR makes this a potentially useful regimen in patients with r/r PTCL.

#### 4.2.3. Romidepsin + Duvelisib

Results of a phase I trial investigating the combination of duvelisib and romidepsin in R/R PTCL and CTCL (*n* = 66) were recently presented [72]. Ten patients received duvelisib (75 mg BID) followed by combined duvelisib + romidepsin (10 mg/m^2^), and the rest began treatment with combination therapy. In the evaluable patients in the PTCL cohort (*n* = 64), an ORR of 58% and a CRR of 42% were observed, with a median PFS of 6.9 months. Among responders, 43% were able to proceed to allo-SCT. Interestingly, patients that began duvelisib monotherapy had a relatively high rate of transaminitis (40%) compared with patients starting on combined therapy (8%), suggesting a potential immunomodulatory effect of romidepsin on duvelisib-induced hepatic inflammation, a known side effect of PI3K delta inhibitors. Other side effects included neutropenia (36%), diarrhea (15%), thrombocytopenia (10%), and infection (10%). These results indicate improved responses and deeper responses with combined PI3K/romidepsin inhibition with a reasonable, if not improved, safety profile compared with monotherapy of each.

## 5. Additional Approaches

Additional targeted approaches for the treatment of r/r PTCL are being explored based on PTCL biology and have already yielded novel treatment avenues. A breakdown can be further seen in Table 3. 

### 5.1. CD47 Inhibition

The expression of CD47 on malignant cells suppresses malignant-cell phagocytosis by interaction with signal regulatory protein α (SIRPα) expressed on macrophages and dendritic cells [76,77,78]. CD47 was found to be heterogeneously expressed in T-cell-lymphoma cell lines [79]. Magrolimab is an investigational anti-CD47 monoclonal antibody (HU5F9-G4) that facilitates macrophage checkpoint inhibition and enables phagocytosis and the elimination of non-Hodgkin lymphoma cells [80]. Anti-CD47 monoclonal antibodies induced the selective phagocytosis of T-cell lymphoma by macrophages in vitro and in murine patient-derived xenograft or immunocompetent T-cell lymphoma models, although the PTCL-NOS cell line was found to be resistant [79]. A phase I study of TTI-621 (SIRPα-IgG1 Fc), an anti-CD47 checkpoint inhibitor, showed that 2 of 9 (22%) subjects with PTCL (angioimmunoblastic T-cell lymphoma), as well as 1 of 4 (25%) patients with Sézary syndrome and 5 of 19 (26%) patients with mycosis fungoides, showed a response to treatment [64]. Unfortunately, none of the PTCL responses were CRs. Additional pre-clinical studies furthering the understanding of this immune checkpoint blockade in PTCL are warranted.

### 5.2. Ruxolitinib

The frequent activation of the JAK/STAT-pathway has been reported in PTCL subtypes [81]. A phase II multicenter trial (#NCT02974647) investigated Ruxolitinib, an oral JAK1/2 inhibitor, in 45 patients with r/r PTCL and 7 patients with mycosis fungoides [66]. Subjects were recruited into three cohorts, including 21 patients with activating JAK and/or STAT mutations, 15 patients with phosphorylated STAT3 expression in at least 30% of malignant cells identified by immunohistochemistry, and 17 patients that did not fit into the above two categories. The combined clinical benefit rate, defined as the combination of complete and partial response and SD for at least 6 months, was demonstrated in 53% of patients in cohort 1, 45% of patients in cohort 2, and 13% of patients in cohort 3 [66]. The overall combined clinical benefit rate was 35%, with an overall response rate of 25%. The highest clinical benefit rate (4/5; 80%) was seen in T-large granular lymphocytosis and T-prolymphocytic leukemia patients (4/8; 50%), while the lowest was seen in mycosis fungoides patients (1/7; 14%). Significantly, mutations in the JAK/STAT pathway have been identified in up to 50% of T-large granular lymphocytosis cases and 40% of T-prolymphocytic leukemia cases [67]. Observed adverse events were typical for ruxolitinib, including cytopenias and infections [66]. These results demonstrate the promising activity of JAK/STAT inhibitor Ruxolitinib in PTCL cases with the activation of the JAK/STAT pathway. 

### 5.3. ITK Inhibitors

Interleukin-2-inducible kinase (ITK) belongs to the TEC family of kinases that regulate T-cell receptor signaling and T-cell differentiation [82]. High levels of ITK expression and chromosomal translocations involving ITK have been observed in AITL [83,84]. Furthermore, a fusion of ITK and Syk kinase mimicked T-cell receptor signaling and drove tumorigenesis in murine models of PTCLs [85]. ITK inhibitors, in combination with doxorubicin and PI3K inhibitors, demonstrated to be able to kill malignant PTCL cell lines in vitro [86]. CPI-818, an irreversible ITK inhibitor, showed a CR in one of seven patients (14%) and SD in three of seven patients (43%) with r/r PTCL in an ongoing phase 1/1b trial (NCT03952078) [65]. The expansion of the cohort with dosing that achieved a 98% ITK inhibition is ongoing [65].

### 5.4. Nanatinostat

The Epstein-Barr virus (EBV) is associated with up to 90% of AITL and 20% of PTCL-NOS cases [87,88], and EBV positivity is associated with a worse prognosis [89,90]. Histone deacetylase inhibitors (HDACs) can induce the expression of lytic EBV protein kinase BGLF4 in latently infected EBV-positive malignant cells [91]. BGLF4 can, in turn, activate anti-viral agent Ganciclovir by phosphorylation, making EBV-infected cells susceptible to anti-viral treatment [92]. A phase 1b/2 VT3996-201 study combined the HDAC inhibitor nanatinostat with valganciclovir for the treatment of histologically confirmed EBV-positive r/r lymphomas [68]. The study enrolled a total of 15 T/NK non-Hodgkin lymphoma subjects: 5 PTCL-NOS, 1 cutaneous T-cell lymphoma, and 9 extranodal NK-/T-cell lymphoma patients. In total, 9 of 15 patients (60%) demonstrated objective response, while 4 (27%) showed a CR, and 2 subjects were removed from the study to proceed to autologous stem-cell transplant [68]. Grade 3–4 adverse events included cytopenias, with only one patient having to discontinue therapy [68]. The final study results warrant further evaluation of Nanatinostat in combination with Ganciclovir or Valganciclovir in T-cell lymphomas.

## 6. Conclusions and Future Perspectives

PTCLs are a group of rare lymphomas with poor outcomes, especially for patients with relapsed and refractory diseases. While many patients will have relapsed/refractory disease to primary treatment, many new treatment options have been developed to change the game for patients with relapsed and refractory PTCLs. The early data from the Primo trial are compelling, with an ORR of 50%, including a 33% CR, in a highly relapsed/refractory population of patients. These results led the NCCN to recommend duvelisib for r/r PTCL. Further, early results using valemetostat demonstrated similar responses, though the outcomes from the phase II Valentine-PTCL01 study have yet to be reported. Importantly, new combinations of previously approved agents, such as romidepsin + 5-azacytidine, bendamustine + BV, and romidepsin + duvelisib, showed promising ORRs that require further investigation. With the arrival of numerous new therapeutic agents and combinations, patients with relapsed/refractory PTCL have new hope for durable responses and improved survival in this devastating disease.

## Figures and Tables

**Table 1:** PTCL Subtypes Distribution and Median Age at Diagnosis [2,4].

PTCL Subtype	Median Age at Diagnosis (Y)	Worldwide Distribution (%)	North American Distribution (%)	European Distribution (%)	Asian Distribution (%)
PTCL-NOS	60	25.6	34.4	34.3	22.4
AITL	65	19.5	16.0	28.7	17.9
ALK − ALCL	58	5.5	16.0	9.4	2.6
ALK + ALCL	34	6.6	7.8	6.4	3.2
NKTCL	52	10.4	5.1	4.3	22.4
ATLL	62	9.6	2.0	1.0	25.0

**Table 2:** Current Standard of Care.

Drug	Regimen	Median PFS	ORR/CR (%)	Most Common Adverse Effects (%)	Patient Considerations	Evidence
Gemcitabine	1200 mg/m^2^ on days 1, 8, and 15 of a 28 day cycle for 3–6 cycles	Unknown	50–70/23	Thrombocytopenia (46) Neutropenia (38.5) Transaminitis (36)	None	Zinzani et al. [18]
Romidepsin	14 mg/m^2^ on days 1, 8, and 15 of 28 day cycles	4 months	25–38/15–18	Nausea (51) Leukopenia (47) Thrombocytopenia (47) Granulocytopenia (45)	Patients with significant cardiac abnormalities were excluded	Piekarz et al. [19] Coiffier et al. [19]
Pralatrexate	30 mg/m^2^/week for 6 weeks followed by 1 week of rest (7-week cycle). Continued until	3.5 months	29/11	Mucositis (79) Nausea (46) Thrombocytopenia (45) Anemia (38)	Administration of folic acid, B12, and leucovorin needed to prevent AEs	O’Connor et al. [20]
Bendamustine	90–120 mg/m^2^ D1-2 of 21 day cycles, for up to six cycles,	3.6 months	50/28	Neutropenia (56) * Thrombocytopenia (38) * Infection (20) *	None	Damaj et al. [21]
ICE	Every 15–22 days for 2–3 cycles. Ifosfamide 5 g/m^2^ on day 2, carboplatin administered on day 2, and dosed to AUC5, and etoposide 100 mg/m^2^ on days 1 to 3	2 months	20/7	Thrombocytopenia (100) Anemia (100) Leukopenia (100) Nausea (28)	Used for salvage therapy to proceed to HDT/ASCT	Mikesch et al. [22]
Brentuximab Vedotin	120 mg/m^2^ on days 1 and 2 every 3 weeks for 6 cycles	3.6 months	50/28	Neutropenia (30) * Thrombocytopenia (24) * Infections (20) *		Damaj et al. [21]

*: ≥ Grade 3.

**Table 3:** Single Agent Investigational Drugs.

Drug	Regimen	Median PFS	ORR/CR (%)	Most Common Adverse Effects (%)	Patient Considerations	Evidence
MEDI-570	3 + 3 study design; IV infusion every 3 weeks for 12 cycles	Unknown	24/0	Decreased CD4 T-Cell count (12) Hypophosphatemia (6) Infusion Reaction (6) Thrombocytopenia (6)	Patients were heavily pre-treated with median 7.5 prior treatments; Study is continuing enrollment in the expansion phase	Chavez et al. [62]
Duvelisib	75 mg BID for 2 months followed by 25 mg BID	Currently unknown	50/32	Neutropenia (39) * Transaminitis (22–24) * Rash (8) * Lymphopenia (8) *	None	Brammer et al. [58]
Valemetostat	200 mg daily on 28 day cycles	13 months	48–55/20–24	Thrombocytopenia (59) Dysgeusia (51) Anemia (37) Neutropenia (35) Alopecia (32)	None	Yoshimitsu et al. [59] Ishitsuka et al. [60]
Romidepsin	14 mg/m^2^ on days 1, 8, and 15 of 28 day cycle for 6 cycles	29 Months	25/15	Nausea (59) Infection (55) Fatigue (55) Thrombocytopenia (41)	Patients with significant cardiac abnormalities require close monitoring	Coiffier et al. [34] Coiffier et al. [33]
Belinostat	1 g/m^2^ on days 1–5 of 21 day cycle for as long as tolerated	1.6 months	25.8/10.8	Nausea (42) Fatigue (37) Pyrexia (35) Thrombocytopenia (16)	Dose reduce in patients homozygous for UGT1A1*28 allele	O’Connor et al. [35]
TTI-621	3 + 3 dose escalation schema	Unknown	22/0	Infusion reaction (43) Thrombocytopenia (26) Chills (18) Anemia (13)	Can potentially be combined with Rituximab or Nivolumab	Ansell et al. [64]
Cpi-818	Dose escalation of 100, 200, 400, 600 mg BID for up to 16 21-day cycles	Unknown	14/14	Fatigue (16) Nausea (11) Rash (11)	None	Khodadoust et al. [65]
Ruxolitinib	20 mg BID on 28 day cycles until progression or toxicity	2.8 months	25/6	Anemia (28) Neutropenia (19) Thrombocytopenia (17) Diarrhea (13%)	None	Moskowitz et al. [66] Pinter-Brown [67]
Nanatinostat with Valganciclovir	Nstat 20mg daily 4 days/week and VGCV 900mg daily in 28-day cycles until progression or toxicity	Unknown	40/19	Nausea (38) Neutropenia (34) Thrombocytopenia (34) Constipation (31)	For patients that are EBV+	Haverkos et al. [68]

*: ≥ Grade 3.

**Table 4:** Combination Investigational Drugs.

Drug	Regimen	Median PFS	ORR/CR (%)	Most Common Adverse Effects (%)	Patient Considerations	Evidence
Bendamustine and Brentuximab Vedotin	Bendamustine 70 mg/m^2^ on day 1 and 2 of and BV 1.8 mg/kg on day 1 of 21 day cycle	8.3 months	71/51	Unknown	Significant neutropenia; recommend q28 day cycles	Bouabdallah et al. [69]
Romidepsin and 5-Azacytadine	Romidepsin 14 mg/m^2^ days 8, 15, 22 and 5-Azacytadine 300 mg days 1–14 of a 35 day cycle	2.3 months	61/42	Thrombocytopenia (72) Neutropenia (68) Nausea (68) Hyperglycemia (60)	Improved responses in patients with tTFH phenotypes.	Falchi et al. [70]
Romidepsin and Pralatrexate	Romidepsin 12 mg/m^2^ and Pralatrexate 25 mg/m^2^ every other week	3.7 months	71/28	Nausea (66) Fatigue (52) Thrombocytopenia (35) Oral Mucositis (33)	none	Amengual et al. [71]
Romidepsin and Duvelisib	Romidepsin 10 mg/m^2^ and Duvelisib 75 mg BID	6.9 months	58/42	Nausea (73) Thrombocytopenia (57) Fatigue (54) Transaminitis (27–33)	None	Iyer et al. [72]
Nanatinostat with Valganciclovir	Nstat 20 mg daily 4 days/week and VGCV 900mg daily in 28-day cycles until progression or toxicity	Unknown	40/19	Nausea (38) Neutropenia (34) Thrombocytopenia (34) Constipation (31)	For patients that are EBV+	Haverkos et al. [68]

## Data Availability

Not applicable.

## References

[1] Swerdlow S.H., Campo E., Pileri S.A., Harris N.L., Stein H., Siebert R., Advani R., Ghielmini M., Salles G.A., Zelenetz A.D. (2016). The 2016 revision of the World Health Organization classification of lymphoid neoplasms. Blood.

[2] Vose J., Armitage J., Weisenburger D., Project I.T.-C.L. (2008). International peripheral T-cell and natural killer/T-cell lymphoma study: Pathology findings and clinical outcomes. J. Clin. Oncol..

[3] Anderson J.R., Armitage J.O., Weisenburger D.D. (1998). Epidemiology of the non-Hodgkin’s lymphomas: Distributions of the major subtypes differ by geographic locations. Non-Hodgkin’s Lymphoma Classification Project. Ann. Oncol..

[4] Moskowitz A.J., Lunning M.A., Horwitz S.M. (2014). How I treat the peripheral T-cell lymphomas. Blood.

[5] Zain J.M. (2019). Aggressive T-cell lymphomas: 2019 updates on diagnosis, risk stratification, and management. Am. J. Hematol..

[6] Ellin F., Landström J., Jerkeman M., Relander T. (2014). Real-world data on prognostic factors and treatment in peripheral T-cell lymphomas: A study from the Swedish Lymphoma Registry. Blood.

[7] Savage K.J., Chhanabhai M., Gascoyne R.D., Connors J.M. (2004). Characterization of peripheral T-cell lymphomas in a single North American institution by the WHO classification. Ann. Oncol..

[8] Carson K.R., Horwitz S.M., Pinter-Brown L.C., Rosen S.T., Pro B., Hsi E.D., Federico M., Gisselbrecht C., Schwartz M., Bellm L.A. (2017). A prospective cohort study of patients with peripheral T-cell lymphoma in the United States. Cancer.

[9] Horwitz S., O’Connor O.A., Pro B., Illidge T., Fanale M., Advani R., Bartlett N.L., Christensen J.H., Morschhauser F., Domingo-Domenech E. (2019). Brentuximab vedotin with chemotherapy for CD30-positive peripheral T-cell lymphoma (ECHELON-2): A global, double-blind, randomised, phase 3 trial. Lancet.

[10] Rogers A.M., Brammer J.E. (2020). Hematopoietic Cell Transplantation and Adoptive Cell Therapy in Peripheral T Cell Lymphoma. Curr. Hematol. Malig. Rep..

[11] Reimer P., Rüdiger T., Geissinger E., Weissinger F., Nerl C., Schmitz N., Engert A., Einsele H., Müller-Hermelink H.K., Wilhelm M. (2009). Autologous stem-cell transplantation as first-line therapy in peripheral T-cell lymphomas: Results of a prospective multicenter study. J. Clin. Oncol..

[12] Elstrom R.L., Martin P., Hurtado Rua S., Shore T.B., Furman R.R., Ruan J., Pearse R.N., Coleman M., Mark T., Leonard J.P. (2012). Autologous stem cell transplant is feasible in very elderly patients with lymphoma and limited comorbidity. Am. J. Hematol..

[13] Network NCC (2020). T-Cell Lymphomas. NCCN Clinical Practice Guidelines.

[14] Corradini P., Dodero A., Farina L., Fanin R., Patriarca F., Miceli R., Matteucci P., Bregni M., Scimè R., Narni F. (2007). Allogeneic stem cell transplantation following reduced-intensity conditioning can induce durable clinical and molecular remissions in relapsed lymphomas: Pre-transplant disease status and histotype heavily influence outcome. Leukemia.

[15] D’Amore F., Relander T., Lauritzsen G.F., Jantunen E., Hagberg H., Anderson H., Holte H., Österborg A., Merup M., Brown P. (2012). Up-front autologous stem-cell transplantation in peripheral T-cell lymphoma: NLG-T-01. J. Clin. Oncol..

[16] Mak V., Hamm J., Chhanabhai M., Shenkier T., Klasa R., Sehn L.H., Villa D., Gascoyne R.D., Connors J.M., Savage K.J. (2013). Survival of patients with peripheral T-cell lymphoma after first relapse or progression: Spectrum of disease and rare long-term survivors. J. Clin. Oncol..

[17] Chihara D., Fanale M.A., Miranda R.N., Noorani M., Westin J.R., Nastoupil L.J., Hagemeister F.B., Fayad L.E., Romaguera J.E., Samaniego F. (2017). The survival outcome of patients with relapsed/refractory peripheral T-cell lymphoma-not otherwise specified and angioimmunoblastic T-cell lymphoma. Br. J. Haematol..

[18] Zinzani P.L., Venturini F., Stefoni V., Fina M., Pellegrini C., Derenzini E., Gandolfi L., Broccoli A., Argnani L., Quirini F. (2010). Gemcitabine as single agent in pretreated T-cell lymphoma patients: Evaluation of the long-term outcome. Ann. Oncol..

[19] Piekarz R.L., Frye R., Prince H.M., Kirschbaum M.H., Zain J., Allen S.L., Jaffe E.S., Ling A., Turner M., Peer C.J. (2011). Phase 2 trial of romidepsin in patients with peripheral T-cell lymphoma. Blood.

[20] O’Connor O.A., Pro B., Pinter-Brown L., Bartlett N., Popplewell L., Coiffier B., Lechowicz M.J., Savage K.J., Shustov A.R., Gisselbrecht C. (2011). Pralatrexate in patients with relapsed or refractory peripheral T-cell lymphoma: Results from the pivotal PROPEL study. J. Clin. Oncol..

[21] Damaj G., Gressin R., Bouabdallah K., Cartron G., Choufi B., Gyan E., Banos A., Jaccard A., Park S., Tournilhac O. (2013). Results from a prospective, open-label, phase II trial of bendamustine in refractory or relapsed T-cell lymphomas: The BENTLY trial. J. Clin. Oncol..

[22] Mikesch J.H., Kuhlmann M., Demant A., Krug U., Thoennissen G.B., Schmidt E., Kessler T., Schliemann C., Pohlen M., Mohr M. (2013). DexaBEAM versus ICE salvage regimen prior to autologous transplantation for relapsed or refractory aggressive peripheral T cell lymphoma: A retrospective evaluation of parallel patient cohorts of one center. Ann. Hematol..

[23] Zhang P., Zhang M. (2020). Epigenetic alterations and advancement of treatment in peripheral T-cell lymphoma. Clin. Epigenet..

[24] Ma H., O’Connor O.A., Marchi E. (2019). New directions in treating peripheral T-cell lymphomas (PTCL): Leveraging epigenetic modifiers alone and in combination. Expert Rev. Hematol..

[25] Ye Y., Ding N., Mi L., Shi Y., Liu W., Song Y., Shu S., Zhu J. (2021). Correlation of mutational landscape and survival outcome of peripheral T-cell lymphomas. Exp. Hematol. Oncol..

[26] Moskowitz A.J., Horwitz S.M. (2017). Targeting histone deacetylases in T-cell lymphoma. Leuk Lymphoma.

[27] Kim H.J., Bae S.C. (2011). Histone deacetylase inhibitors: Molecular mechanisms of action and clinical trials as anti-cancer drugs. Am. J. Transl. Res..

[28] Sanaei M., Kavoosi F. (2019). Histone Deacetylases and Histone Deacetylase Inhibitors: Molecular Mechanisms of Action in Various Cancers. Adv. Biomed. Res..

[29] Xu W.S., Parmigiani R.B., Marks P.A. (2007). Histone deacetylase inhibitors: Molecular mechanisms of action. Oncogene.

[30] Mrakovcic M., Kleinheinz J., Fröhlich L.F. (2019). p53 at the Crossroads between Different Types of HDAC Inhibitor-Mediated Cancer Cell Death. Int. J. Mol. Sci..

[31] Yan W., Liu S., Xu E., Zhang J., Zhang Y., Chen X. (2013). Histone deacetylase inhibitors suppress mutant p53 transcription via histone deacetylase 8. Oncogene.

[32] Piekarz R.L., Robey R., Sandor V., Bakke S., Wilson W.H., Dahmoush L., Kingma D.M., Turner M.L., Altemus R., Bates S.E. (2001). Inhibitor of histone deacetylation, depsipeptide (FR901228), in the treatment of peripheral and cutaneous T-cell lymphoma: A case report. Blood.

[33] Coiffier B., Pro B., Prince H.M., Foss F., Sokol L., Greenwood M., Caballero D., Borchmann P., Morschhauser F., Wilhelm M. (2012). Results from a pivotal, open-label, phase II study of romidepsin in relapsed or refractory peripheral T-cell lymphoma after prior systemic therapy. J. Clin. Oncol..

[34] Coiffier B., Pro B., Prince H.M., Foss F., Sokol L., Greenwood M., Caballero D., Morschhauser F., Wilhelm M., Pinter-Brown L. (2014). Romidepsin for the treatment of relapsed/refractory peripheral T-cell lymphoma: Pivotal study update demonstrates durable responses. J. Hematol. Oncol..

[35] O’Connor O.A., Horwitz S., Masszi T., Van Hoof A., Brown P., Doorduijn J., Hess G., Jurczak W., Knoblauch P., Chawla S. (2015). Belinostat in Patients With Relapsed or Refractory Peripheral T-Cell Lymphoma: Results of the Pivotal Phase II BELIEF (CLN-19) Study. J. Clin. Oncol..

[36] Johnston P.B., Cashen A.F., Nikolinakos P.G., Beaven A.W., Barta S.K., Bhat G., Hasal S.J., De Vos S., Oki Y., Deng C. (2021). Belinostat in combination with standard cyclophosphamide, doxorubicin, vincristine and prednisone as first-line treatment for patients with newly diagnosed peripheral T-cell lymphoma. Exp. Hematol. Oncol..

[37] Bachy E., Camus V., Thieblemont C., Sibon D., Casasnovas R.O., Ysebaert L., Damaj G., Guidez S., Pica G.M., Kim W.S. (2022). Romidepsin Plus CHOP Versus CHOP in Patients With Previously Untreated Peripheral T-Cell Lymphoma: Results of the Ro-CHOP Phase III Study (Conducted by LYSA). J. Clin. Oncol..

[38] O’Connor O.A., Horwitz S., Hamlin P., Portlock C., Moskowitz C.H., Sarasohn D., Neylon E., Mastrella J., Hamelers R., Macgregor-Cortelli B. (2009). Phase II-I-II study of two different doses and schedules of pralatrexate, a high-affinity substrate for the reduced folate carrier, in patients with relapsed or refractory lymphoma reveals marked activity in T-cell malignancies. J. Clin. Oncol..

[39] Hong J.Y., Yoon D.H., Yoon S.E., Kim S.J., Lee H.S., Eom H.S., Lee H.W., Shin D.Y., Koh Y., Yoon S.S. (2019). Pralatrexate in patients with recurrent or refractory peripheral T-cell lymphomas: A multicenter retrospective analysis. Sci. Rep..

[40] Zhu J., Yeoh E.M., Maeda Y., Tobinai K. (2020). Efficacy and safety of single-agent pralatrexate for treatment of angioimmunoblastic T-cell lymphoma after failure of first line therapy: A pooled analysis. Leuk. Lymphoma.

[41] Shustov A.R., Mehta A., Sokol L., Carvajal L.A., Guerlavais V., Samant M., Narasimhan N., Sutton D., Annis D.A., Pinchasik D. (2018). Pseudoprogression (PsP) in Patients with Peripheral T-Cell Lymphoma (PTCL) Treated with the Novel Stapled Peptide ALRN-6924, a Dual Inhibitor of MDMX and MDM2. Blood.

[42] Zinzani P.L., Magagnoli M., Bendandi M., Orcioni G.F., Gherlinzoni F., Albertini P., Pileri S.A., Tura S. (1998). Therapy with gemcitabine in pretreated peripheral T-cell lymphoma patients. Ann. Oncol..

[43] Dong M., He X.H., Liu P., Qin Y., Yang J.L., Zhou S.Y., Yang S., Zhang C.G., Gui L., Zhou L.Q. (2013). Gemcitabine-based combination regimen in patients with peripheral T-cell lymphoma. Med. Oncol..

[44] Arkenau H.T., Chong G., Cunningham D., Watkins D., Sirohi B., Chau I., Wotherspoon A., Norman A., Horwich A., Matutes E. (2007). Gemcitabine, cisplatin and methylprednisolone for the treatment of patients with peripheral T-cell lymphoma: The Royal Marsden Hospital experience. Haematologica.

[45] Van de Wyngaert Z., Coppo P., Cervera P., Fabiani B., Lemonnier M.P., Corre E., Marjanovic Z., Aoudjhane M., Mohty M., Duléry R. (2021). Combination of brentuximab-vedotin and ifosfamide, carboplatin, etoposide in relapsed/refractory peripheral T-cell lymphoma. Eur. J. Haematol..

[46] Horwitz S.M., Ansell S., Ai W.Z., Barnes J., Barta S.K., Clemens M.W., Dogan A., Goodman A.M., Goyal G., Guitart J. (2020). NCCN Guidelines Insights: T-Cell Lymphomas, Version 1.2021. J. Natl. Compr. Cancer Netw..

[47] Sabattini E., Pizzi M., Tabanelli V., Baldin P., Sacchetti C.S., Agostinelli C., Zinzani P.L., Pileri S.A. (2013). CD30 expression in peripheral T-cell lymphomas. Haematologica.

[48] Karube K., Kakimoto Y., Tonozuka Y., Ohshima K. (2021). The expression of CD30 and its clinico-pathologic significance in peripheral T-cell lymphomas. Expert Rev. Hematol..

[49] Savage K.J., Harris N.L., Vose J.M., Ullrich F., Jaffe E.S., Connors J.M., Rimsza L., Pileri S.A., Chhanabhai M., Gascoyne R.D. (2008). ALK-anaplastic large-cell lymphoma is clinically and immunophenotypically different from both ALK^+^ ALCL and peripheral T-cell lymphoma, not otherwise specified: Report from the International Peripheral T-Cell Lymphoma Project. Blood.

[50] Bisig B., de Reyniès A., Bonnet C., Sujobert P., Rickman D.S., Marafioti T., Delsol G., Lamant L., Gaulard P., de Leval L. (2013). CD30-positive peripheral T-cell lymphomas share molecular and phenotypic features. Haematologica.

[51] Pro B., Advani R., Brice P., Bartlett N.L., Rosenblatt J.D., Illidge T., Matous J., Ramchandren R., Fanale M., Connors J.M. (2012). Brentuximab vedotin (SGN-35) in patients with relapsed or refractory systemic anaplastic large-cell lymphoma: Results of a phase II study. J. Clin. Oncol..

[52] Pro B., Advani R., Brice P., Bartlett N.L., Rosenblatt J.D., Illidge T., Matous J., Ramchandren R., Fanale M., Connors J.M. (2017). Five-year results of brentuximab vedotin in patients with relapsed or refractory systemic anaplastic large cell lymphoma. Blood.

[53] Horwitz S.M., Advani R.H., Bartlett N.L., Jacobsen E.D., Sharman J.P., O’Connor O.A., Siddiqi T., Kennedy D.A., Oki Y. (2014). Objective responses in relapsed T-cell lymphomas with single-agent brentuximab vedotin. Blood.

[54] Barta S.K., Gong J.Z., Porcu P. (2019). Brentuximab vedotin in the treatment of CD30+ PTCL. Blood.

[55] Stefoni V., Corradini P., Orsucci L., Volpetti S., Argnani L., Dodero A., Pellegrini C., Zinzani P.L. (2020). Brentuximab Vedotin As Single Agent in the Treatment of Relapsed/Refractory CD30 Positive Peripheral T-Cell Lymphoma Patients: A Phase 2 Study of the Fondazione Italiana Linfomi. Blood.

[56] Horwitz S.M., Koch R., Porcu P., Oki Y., Moskowitz A., Perez M., Myskowski P., Officer A., Jaffe J.D., Morrow S.N. (2018). Activity of the PI3K-delta,gamma inhibitor duvelisib in a phase 1 trial and preclinical models of T-cell lymphoma. Blood.

[57] Brammer J.E. (2018). Dual PI3K blockade: PTCL’s Achilles heel?. Blood.

[58] Brammer J.E., Zinzani P.L., Zain J., Mead M., Casulo C., Jacobsen E.D., Gritti G., Litwak D., Cohan D., Katz D.J. (2021). Duvelisib in Patients with Relapsed/Refractory Peripheral T-Cell Lymphoma from the Phase 2 Primo Trial: Results of an Interim Analysis. Blood.

[59] Yoshimitsu M., Izutsu K., Makita S., Nosaka K., Utsunomiya A., Kusumoto S., Morishima S., Tsukasaki K., Kawamata T., Ono T. (2021). Pivotal Phase 2 Study of the EZH1 and EZH2 Inhibitor Valemetostat Tosylate (ds-3201B) in Patients with Relapsed or Refractory Adult T-Cell Leukemia/Lymphoma. Blood.

[60] Ishitsuka K., Izutsu K., Maruyama D., Makita S., Jacobsen E.D., Horwitz S., Kusumoto S., Allen P., Porcu P., Imaizumi Y. (2021). First-in-human study of the ezh1 and ezh2 dual inhibitor valemetostat tosylate (ds-3201b) in patients with relapsed or refractory non-hodgkin lymphomas. Hematol. Oncol..

[61] Foss F.M., Porcu P., Horwitz S.M., Izutsu K., Ishitsuka K., Kato K., Jin J., Du Y., Inoue A. (2021). A Global Phase 2 Study of Valemetostat Tosylate (Valemetostat) in Patients with Relapsed or Refractory (R/R) Peripheral T-Cell Lymphoma (PTCL), Including R/R Afult T-cell Leukemia/Lymphoma (ATL)-Valentine-PTCL01. Blood.

[62] Chavez J.C., Foss F.M., William B.M., Brammer J.E., Smith S.M., Prica A., Zain J.M., Tuscano J.M., Glenn M., Mehta-Shah N. (2021). A Phase I Study of Anti-ICOS Antibody MEDI-570 for Relapsed/refractory (R/R) Peripheral T-Cell Lymphoma (PTCL) and Angioimmunoblastic T-Cell Lymphoma (AITL) (NCI-9930). Blood.

[63] Nicholson S.M., Carlesso G., Cheng L.I., Cook H., DaCosta K., Leininger J., McKeever K., Scott S.W., Taylor D., Streicher K. (2017). Effects of ICOS+ T cell depletion via afucosylated monoclonal antibody MEDI-570 on pregnant cynomolgus monkeys and the developing offspring. Reprod. Toxicol..

[64] Ansell S.M., Maris M.B., Lesokhin A.M., Chen R.W., Flinn I.W., Sawas A., Minden M.D., Villa D., Percival M.M., Advani A.S. (2021). Phase I Study of the CD47 Blocker TTI-621 in Patients with Relapsed or Refractory Hematologic Malignancies. Clin. Cancer Res..

[65] Khodadoust M.S., Feldman T.A., Yoon D.H., Yannakou C.K., Radeski D., Kim Y.H., Mehta-Shah N., Khot A., Wilcox R.A., Kim W.S. (2020). Cpi-818, an Oral Interleukin-2-Inducible T-Cell Kinase Inhibitor, Is Well-Tolerated and Active in Patients with T-Cell Lymphoma. Blood.

[66] Moskowitz A.J., Ghione P., Jacobsen E., Ruan J., Schatz J.H., Noor S., Myskowski P., Vardhana S., Ganesan N., Hancock H. (2021). A phase 2 biomarker-driven study of ruxolitinib demonstrates effectiveness of JAK/STAT targeting in T-cell lymphomas. Blood.

[67] Pinter-Brown L.C. (2021). JAK/STAT: A pathway through the maze of PTCL?. Blood.

[68] Haverkos B.M., Alpdogan O., Baiocchi R., Brammer J.E., Feldman T.A., Capra M., Brem E.A., Nair S.M., Scheinberg P., Pereira J. (2021). Nanatinostat (Nstat) and Valganciclovir (VGCV) in Relapsed/Refractory (R/R) Epstein-Barr Virus-Positive (EBV +) Lymphomas: Final Results from the Phase 1b/2 VT3996-201 Study. Blood.

[69] Bouabdallah K., Aubrais R., Chartier L., Herbaux C., Banos A., Brice P., Ochmann M., Sibon D., Schiano De Colella J.M., Cluzeau T. (2021). Salvage Therapy with Brentuximab-Vedotin and Bendamustine for Patients with Relapsed/Refractory T Cell Lymphoma. a Multicenter and Retrospective Study. Blood.

[70] Falchi L., Ma H., Klein S., Lue J.K., Montanari F., Marchi E., Deng C., Kim H.A., Rada A., Jacob A.T. (2021). Combined oral 5-azacytidine and romidepsin are highly effective in patients with PTCL: A multicenter phase 2 study. Blood.

[71] Amengual J.E., Lichtenstein R., Lue J., Sawas A., Deng C., Lichtenstein E., Khan K., Atkins L., Rada A., Kim H.A. (2018). A phase 1 study of romidepsin and pralatrexate reveals marked activity in relapsed and refractory T-cell lymphoma. Blood.

[72] Iyer S.P., Huen A., Ai W.Z., Jagadeesh D., Lechowicz M.J., Okada C., Feldman T.A., Sundaram S., Alderuccio J.P., Reddy N. (2021). Safety and Efficacy of Tenalisib Given in Combination with Romidepsin in Patients with Relapsed/Refractory T-Cell Lymphoma: Final Results from a Phase I/II Open Label Multi-Center Study. Blood.

[73] Park B.B., Kim W.S., Suh C., Hong J.Y., Yang D.H., Lee W.S., Do Y.R., Koh Y.I., Won J.H., Kim M.K. (2019). A phase II trial of bendamustine, carboplatin, and dexamethasone for refractory or relapsed peripheral T-cell lymphoma (BENCART trial). Leuk. Lymphoma.

[74] Rozati S., Cheng P.F., Widmer D.S., Fujii K., Levesque M.P., Dummer R. (2016). Romidepsin and Azacitidine Synergize in their Epigenetic Modulatory Effects to Induce Apoptosis in CTCL. Clin. Cancer Res..

[75] O’Connor O.A., Falchi L., Lue J.K., Marchi E., Kinahan C., Sawas A., Deng C., Montanari F., Amengual J.E., Kim H.A. (2019). Oral 5-azacytidine and romidepsin exhibit marked activity in patients with PTCL: A multicenter phase 1 study. Blood.

[76] McCracken M.N., Cha A.C., Weissman I.L. (2015). Molecular Pathways: Activating T Cells after Cancer Cell Phagocytosis from Blockade of CD47 “Don’t Eat Me” Signals. Clin. Cancer Res..

[77] Gholamin S., Mitra S.S., Feroze A.H., Liu J., Kahn S.A., Zhang M., Esparza R., Richard C., Ramaswamy V., Remke M. (2017). Disrupting the CD47-SIRPα anti-phagocytic axis by a humanized anti-CD47 antibody is an efficacious treatment for malignant pediatric brain tumors. Sci. Transl. Med..

[78] Tseng D., Volkmer J.P., Willingham S.B., Contreras-Trujillo H., Fathman J.W., Fernhoff N.B., Seita J., Inlay M.A., Weiskopf K., Miyanishi M. (2013). Anti-CD47 antibody-mediated phagocytosis of cancer by macrophages primes an effective antitumor T-cell response. Proc. Natl. Acad. Sci. USA.

[79] Jain S., Van Scoyk A., Morgan E.A., Matthews A., Stevenson K., Newton G., Powers F., Autio A., Louissaint A., Pontini G. (2019). Targeted inhibition of CD47-SIRPα requires Fc-FcγR interactions to maximize activity in T-cell lymphomas. Blood.

[80] Chao M.P., Alizadeh A.A., Tang C., Myklebust J.H., Varghese B., Gill S., Jan M., Cha A.C., Chan C.K., Tan B.T. (2010). Anti-CD47 antibody synergizes with rituximab to promote phagocytosis and eradicate non-Hodgkin lymphoma. Cell.

[81] Manso R., Sánchez-Beato M., González-Rincón J., Gómez S., Rojo F., Mollejo M., García-Cosio M., Menárguez J., Piris M.A., Rodríguez-Pinilla S.M. (2018). Mutations in the JAK/STAT pathway genes and activation of the pathway, a relevant finding in nodal Peripheral T-cell lymphoma. Br. J. Haematol..

[82] Andreotti A.H., Schwartzberg P.L., Joseph R.E., Berg L.J. (2010). T-cell signaling regulated by the Tec family kinase, Itk. Cold Spring Harb. Perspect. Biol..

[83] Attygalle A.D., Feldman A.L., Dogan A. (2013). ITK/SYK translocation in angioimmunoblastic T-cell lymphoma. Am. J. Surg. Pathol..

[84] Agostinelli C., Rizvi H., Paterson J., Shende V., Akarca A.U., Agostini E., Fuligni F., Righi S., Spagnolo S., Piccaluga P.P. (2014). Intracellular TCR-signaling pathway: Novel markers for lymphoma diagnosis and potential therapeutic targets. Am. J. Surg. Pathol..

[85] Pechloff K., Holch J., Ferch U., Schweneker M., Brunner K., Kremer M., Sparwasser T., Quintanilla-Martinez L., Zimber-Strobl U., Streubel B. (2010). The fusion kinase ITK-SYK mimics a T cell receptor signal and drives oncogenesis in conditional mouse models of peripheral T cell lymphoma. J. Exp. Med..

[86] Mamand S., Allchin R.L., Ahearne M.J., Wagner S.D. (2018). Comparison of interleukin-2-inducible kinase (ITK) inhibitors and potential for combination therapies for T-cell lymphoma. Sci. Rep..

[87] Hsi E.D., Said J., Macon W.R., Rodig S.J., Ondrejka S.L., Gascoyne R.D., Morgan E.A., Dorfman D.M., Maurer M.J., Dogan A. (2014). Diagnostic accuracy of a defined immunophenotypic and molecular genetic approach for peripheral T/NK-cell lymphomas. A North American PTCL study group project. Am. J. Surg. Pathol..

[88] Hsi E.D., Horwitz S.M., Carson K.R., Pinter-Brown L.C., Rosen S.T., Pro B., Federico M., Gisselbrecht C., Schwartz M., Bellm L.A. (2017). Analysis of Peripheral T-cell Lymphoma Diagnostic Workup in the United States. Clin. Lymphoma Myeloma Leuk..

[89] Zhao D., Hu S., Zhou D., Zhang Y., Wang W., Zhang W. (2022). Clinical Significance of Plasma Epstein-Barr Virus DNA in Peripheral T-Cell Lymphomas. Acta Haematol..

[90] Kim T.Y., Min G.J., Jeon Y.W., Park S.S., Park S., Shin S.H., Yahng S.A., Yoon J.H., Lee S.E., Cho B.S. (2021). Impact of Epstein-Barr Virus on Peripheral T-Cell Lymphoma Not Otherwise Specified and Angioimmunoblastic T-Cell Lymphoma. Front. Oncol..

[91] Ghosh S.K., Perrine S.P., Williams R.M., Faller D.V. (2012). Histone deacetylase inhibitors are potent inducers of gene expression in latent EBV and sensitize lymphoma cells to nucleoside antiviral agents. Blood.

[92] Meng Q., Hagemeier S.R., Fingeroth J.D., Gershburg E., Pagano J.S., Kenney S.C. (2010). The Epstein-Barr virus (EBV)-encoded protein kinase, EBV-PK, but not the thymidine kinase (EBV-TK), is required for ganciclovir and acyclovir inhibition of lytic viral production. J. Virol..

